# Identification, expression and serological evaluation of the recombinant ATP synthase beta subunit of *Mycoplasma pneumoniae*

**DOI:** 10.1186/1471-2180-10-216

**Published:** 2010-08-11

**Authors:** Hélène Nuyttens, Camille Cyncynatus, Hélène Renaudin, Sabine Pereyre, Cécile Bébéar

**Affiliations:** 1 Laboratoire de Bactériologie EA 3671, Université Victor Segalen Bordeaux and CHU de Bordeaux, Bordeaux, France; 2 InGen BioSciences, Chilly Mazarin, France

## Abstract

**Background:**

*Mycoplasma pneumoniae *is responsible for acute respiratory tract infections (RTIs) common in children and young adults. As *M. pneumoniae *is innately resistant to β-lactams antibiotics usually given as the first-line treatment for RTIs, specific and early diagnosis is important in order to select the right treatment. Serology is the most used diagnostic method for *M. pneumoniae *infections.

**Results:**

In this study, we identified the *M. pneumoniae *ATP synthase beta subunit (AtpD) by serologic proteome analysis and evaluated its usefulness in the development of a serological assay. We successfully expressed and purified recombinant AtpD (rAtpD) protein, which was recognised by serum samples from *M. pneumoniae*-infected patient in immunoblots. The performance of the recombinant protein rAtpD was studied using a panel of serum samples from 103 infected patients and 86 healthy blood donors in an in-house IgM, IgA and IgG enzyme-linked immunosorbent assay (ELISA). The results of this assay were then compared with those of an in-house ELISA with a recombinant C-terminal fragment of the P1 adhesin (rP1-C) and of the commercial Ani Labsystems ELISA kit using an adhesin P1-enriched whole-cell extract. Performances of the rAtpD and rP1-C antigen combination were further assessed by binary logistic regression analysis. We showed that combination of rAtpD and rP1-C discriminated maximally between the patients infected with *M. pneumoniae *(children and adults) and the healthy subjects for the IgM class, performing better than the single recombinant antigens or the commercial whole-cell extract.

**Conclusion:**

These results suggest that AtpD can be used as an antigen for the immunodiagnosis of early and acute *M. pneumoniae *infection in association with adhesin P1, providing an excellent starting point for the development of point-of-care diagnostic assays.

## Background

*Mycoplasma pneumoniae *is a cell wall-less bacterium belonging to the *Mollicutes *class, which invades the human host respiratory epithelium by adhering with a tip-like attachment organelle. Several proteins, including the major surface adhesins P1, P30, P116 and proteins HMW1 to HMW3, as well as proteins A, B and C, interact to constitute this tip-like attachment organelle [1-5]. *M. pneumoniae *causes atypical pneumonia and other respiratory tract infections (RTIs) such as tracheobronchitis, and is responsible for up to 20% of all cases of community-acquired pneumonia, especially among school-aged children and young adults [6,7].

*M. pneumoniae *is intrinsically resistant to beta-lactams antibiotics usually given as the first-line treatment of RTIs. Macrolides and related antibiotics represent the treatment of choice for *M. pneumoniae *respiratory infections. Therefore, an early and specific diagnosis is necessary to give the patient the correct antibiotic treatment.

Serology, including the complement fixation test (CFT) and different enzyme-linked immunosorbent assays (ELISA), is the most common laboratory method used for the diagnosis of *M. pneumoniae *infection although culture methods and PCR are also performed. The CFT may have limited value because it also measures antibodies derived from earlier infections and antibodies to *M. pneumoniae *glycolipid antigens; thus, it can react with antigens of different origins [7]. Previous studies comparing the CFT to the PCR detection of *M. pneumoniae*, however found good sensitivity and specificity for the CFT [8,9]. Many ELISA-based assays, using protein extracts, membrane preparations, glycolipid extracts or whole cell lysates have been developed for the detection of *M. pneumoniae *infection [8]. In particular, good sensitivity has been observed for assays with P1 adhesin-enriched extracts [8,10,11]. In a study by Beersma *et al. *[8], 12 commercial serologic assays for *M. pneumoniae *specific immunoglobulins M and G and the CFT were evaluated with PCR used as the "gold standard". The IgM assay that showed the best sensitivity and specificity were from the Ani Labsystems (77% and 92%, respectively) corresponding to P1-enriched extracts. Other studies have reported the superiority of assays using the P1-enriched antigen on the basis of a comparison of the strength of the immune response [9,11,12]. Nonetheless in the same studies, high IgG seroprevalence has been observed in the control sera ranging from 36% (Virotech assay) to 93% (Ani Labsystems assay). The variability of the ELISA results observed in these studies suggests the need for improved sensitivity and specificity among commercialised serological assays used to detect *M. pneumoniae *infection [8].

Recently, many studies have reported great interest in using a recombinant protein corresponding to the C-terminal portion of the P1 adhesin, which has been described as the immunodominant antigen in *M. pneumoniae *[2,13-17]. Antigenic properties of recombinant proteins P116 and P30 have also been shown [15,18,19]. A combination of frequently recognized antigens could be useful for diagnostic purposes. Thus, the identification of antigenic *M. pneumoniae *RTI-related proteins appears to be a prerequisite for the development of serological test kits based on recombinant antigens.

In this study, we used serologic proteome analysis of *M. pneumoniae *M129 total extracts to simultaneously identify candidate antigens inducing an antibody response [20]. We focused on the ATP synthase beta subunit (AtpD) of *M. pneumoniae *as it was likely to generate an antibody response in *M. pneumoniae*-infected children and adults at an early stage of infection. The *atpD *gene (*mpn598*) contains an open reading frame of 1,428 nucleotides and encodes a protein of 475 amino acids, with a calculated molecular weight of 52,486 Da [21-23]. It was cloned and expressed in *E. coli *to obtain recombinant protein. We then compared the serological performance of this antigen with a previously described recombinant C-terminal fragment of the P1 adhesin (rP1-C) [2,13,15], using in-house IgM, IgA and IgG ELISAs and the commercial Ani Labsystems ELISA that uses an adhesin P1-enriched whole extract. We further evaluated the performance of the combination rAtpD and rP1-C IgM by binary logistic regression analysis to compare results between the recombinant antigens, either alone or together, and the enriched whole extract.

## Results

### Identification of the AtpD antigen by serologic proteome analysis

**Figure 1:**
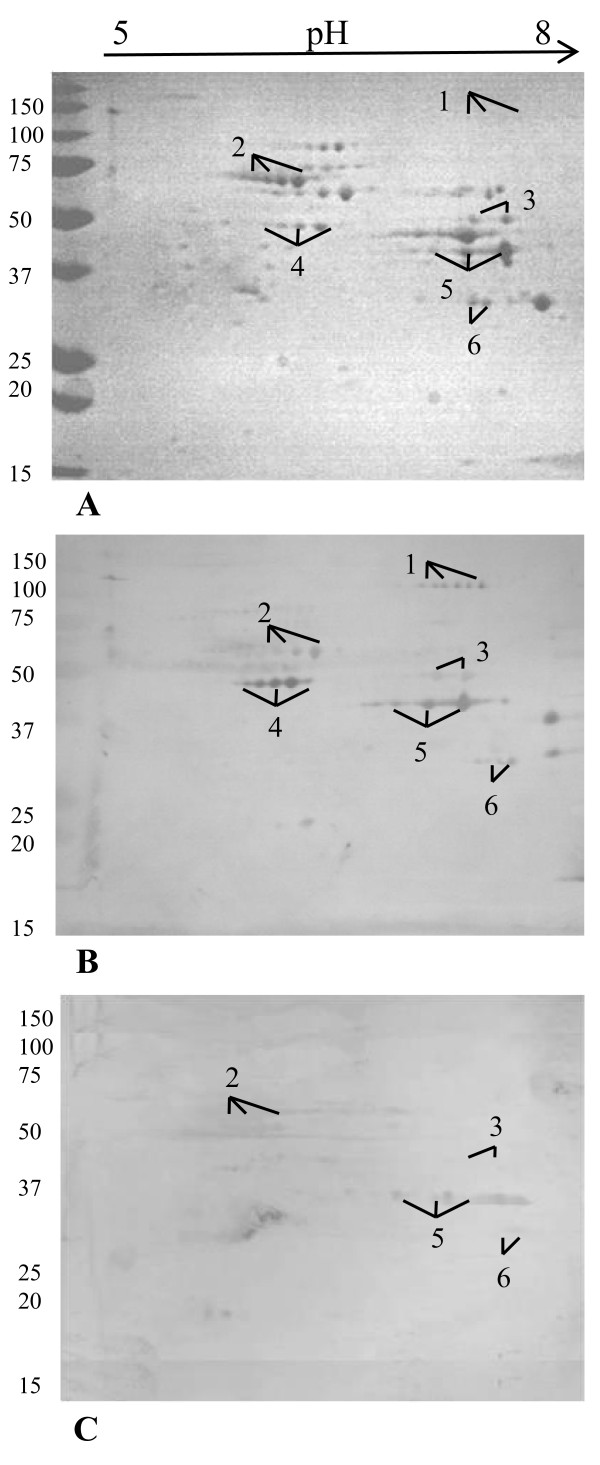
**2D-E profile of *M. pneumoniae *M129 total extract and immunoblots**. 2D-E profile of total extracts (A) and immunoblots probed with 10 serum samples from RTI patients infected with *M. pneumoniae *(B) or 10 serum samples from healthy blood donors (C). Labelled spots represent the *M. pneumoniae *antigenic proteins that were detected with serum samples from the study population. The gel spots were encoded using a protein number (Table 1), which was assigned based on their similar locations on different gels/membranes.

The total protein fraction obtained from the *M. pneumoniae *M129 strain was separated by two dimensional gel electrophoresis (2D-E) (Fig. 1A) and the staining pattern of the 2D immunoblots was probed with 10 different serums samples from patients with RTIs (Fig. 1B) or healthy blood donors (Fig. 1C). The protein identities of six spots that were detected by at least one of the serum samples from the 10 RTI patients were determined using MALDI-TOF mass spectrometry following in-gel tryptic digestion (Table 1). Of the six proteins identified, four (P1 protein, enolase, the ATP synthase beta subunit and the pyruvate dehydrogenase beta subunit) were highly detected by serum samples from patients (Fig. 1B), but only two proteins, the P1 protein and the ATP synthase beta subunit, showed no reactivity with serum samples from healthy blood donors (Fig. 1C).

**Table 1:** Antigenic proteins^a ^identified in this study

Spot no.^b^	Gene no.^c^	Protein name	No. of matching peptides	Sequence coverage (%)	pI^d^	Mass (Da)^d^
1	MPN141	Adhesin P1	24	16	6.4	176.2

2	MPN573	Heat shock protein GroEl	30	59	5.5	58.1

3	MPN606	Enolase	14	45	6.1	49.3

4	MPN598	ATP synthase beta subunit	29	80	5.4	52.3

5	MPN392	Pyruvate dehydrogenase E1 β subunit	21	57	6.2	40.6

6	MPN025	Fructose bisphosphate aldolase	10	44	6.4	31.3

^a ^Antigenic proteins were separated by 2-DE, and their identities were determined by peptide mass fingerprinting.

^b ^Spot numbers are shown in Fig. 1.

^c ^Gene number in *M. pneumoniae *M129.

^d ^Values for pI and mass are theoretical values from the deduced amino acid sequence of the identified gene and open reading frame, respectively.

### Expression, characterization and purification of rAtpD and rP1-C proteins

**Figure 2:**
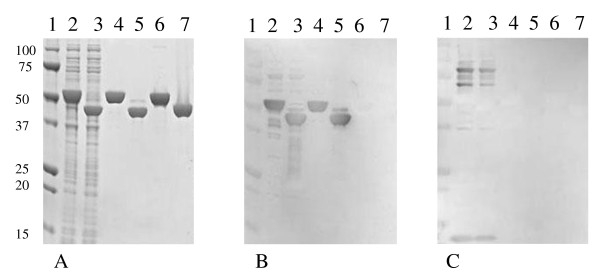
**SDS-PAGE (A) and western blot analysis (B, C) of expressed and purified recombinant proteins**. (A) SDS-PAGE analysis of the expression of rAtpD and rP1-C in *E. coli *extracts (lanes 2 and 3 for rAtpD and rP1-C, respectively) and of the purified recombinant proteins (lanes 4 and 5 for rAtpD and rP1-C, respectively). Two irrelevant his-tagged proteins of the same mass as rAtpD (lane 6) and rP1-C (lane 7) were purified and included in the study. (B, C) Western blot analysis of the expression of rAtpD and rP1-C in *E. coli *extracts (lanes 2 and 3, respectively), of the purified recombinant proteins (lanes 4 and 5 for rAtpD and rP1-C, respectively) and of the two irrelevant his-tagged proteins of the same mass as rAtpD (lane 6) and rP1-C (lane 7) with a pool of 10 serum samples from *M. pneumoniae*-positive patients (B) and with a pool of 10 healthy blood donors (C). Lanes: 1, standard protein marker; 2, induced rAtpD (about 50 kDa); 3, induced rP1-C (about 40 kDa); 4, purified rAtpD; 5, purified rP1-C; 6, irrelevant his-tagged protein of the same mass as rAtpD; 7, irrelevant his-tagged protein of the same mass as r P1-C. The numbers on the left indicate molecular masses (in kDa).

The *atpD *gene and the C-terminal fragment of *p1 *were amplified by PCR and expressed in *E. coli *BL21 (DE3) cells after cloning into the expression vector pDEST 17. These proteins were further purified by affinity column and ion exchange chromatography. The expression and purification of the rAtpD and rP1-C proteins were analysed by sodium dodecyl sulfate-polyacrylamide gel electrophoresis (SDS-PAGE) and western blot (Fig. 2). Two irrelevant purified his-tagged recombinant proteins of the same mass as rAtpD (Fig. 2, lane 6) and rP1-C (Fig. 2, lane 7) were included in the analysis. Both rAtpD and rP1-C were successfully expressed in *E. coli *(Fig. 2A, lane 2 for rAtpD and lane 3 for rP1 -C) and purified with a purity estimated to be 100% by densitometry (Fig. 2A, lane 4 for rAtpD and lane 5 for rP1-C). The apparent molecular masses of rAtpD and rP1-C were about 40 and 50 kDa, respectively, in agreement with the theoretical values.

The rAtpD and rP1-C proteins were both recognised by pooled *M. pneumoniae*-positive serum samples (Fig. 2B, lanes 2 and 4 for rAtpD, lanes 3 and 5 for rP1-C), but not by healthy blood donors (Fig. 2C, lanes 2 and 4 for rAtpD, lanes 3 and 5 for rP1-C). The two irrelevant proteins were not recognised by serum samples from either patients or healthy blood donors (Fig. 2B and 2C, lanes 6 and 7). These results show that *M. pneumoniae-*infected patients have circulating anti-AtpD and anti-rP1 -C antibodies, thereby confirming that these two recombinant proteins are antigenic.

### rAtpD and rP1-C ELISA tests

Serum samples from 103 patients (54 children, 49 adults) with *M. pneumoniae *RTIs and 86 healthy blood donors were screened for anti-*M. pneumoniae *IgM, IgA and IgG antibodies using an in-house ELISA with rAtpD and rP1-C (Tables 2 and 3). We set positive criteria as a value above the cut-off determined by receiver operating characteristics curve (ROC) analysis. The cut-off values of the IgM, IgA and IgG ELISA tests were determined as an absorbance value of 0.4, 0.2, and 0.4, respectively, for rAtpD, and of 0.4, 0.5 and 0.4, respectively for rP1-C. The rAtpD protein demonstrated a higher discriminating score (0.842 ≤ area under curve (AUC) ≤ 0.943) than rP1-C for all of the Ig classes in children and adults (Tables 2 and 3). Among the 54 serum samples from children tested, 38 (70%) showed a high IgM titre compared with rAtpD, whereas 30 (56%) were IgA-positive and 42 (78%) were IgG-positive. Serum samples from 38 (70%) children were positive for IgM against the rP1-C protein, whereas 27 (50%) and 37 (69%) were IgA- and IgG-positive, respectively (Table 2). Out of the 49 serum samples from adults infected with *M. pneumoniae*, 33 (67%) and 22 (45%) tested positive for IgM antibodies against the rAtpD and rP1-C proteins, respectively. Of these samples, 32 (65%) and 27 (55%) reacted with the rAtpD and rP1-C proteins, respectively, for the IgA class, whereas 30 (61%) and 22 (45%) were IgG-positive for the rAtpD and rP1-C proteins, respectively (Table 3). Specificity values ranging from 90% to 97% were found for IgM, IgA and IgG rAtpD and rP1-C protein ELISAs, meaning that no more than 3% to 10% of the serum samples from healthy donors had absorbance values above the cut-off (Tables 2 and 3).

**Table 2:** Performance of the rAtpD, rP1-C ELISAs and the Ani Labsystems kit in children

Ig class	Type of test	No. of positive sera in		Sensitivity (95% CI)	Specificity (95% CI)	AUC
		Patients^a ^(54)	Controls^b ^(86)			
M	rAtpD	38	3	70% (58%-83%)	97% (93%-100%)	0.923

M	rP1-C	38	9	70% (58%-83%)	90% (83%-96%)	0.897

M	rAtpD-rP1-C	40	5	74% (60%-80%)	94% (89%-99%)	0.925

M	Ani Labsystems	39	7	72% (60%-84%)	92% (81%-97%)	0.824

A	rAtpD	30	5	56% (42%-69%)	94% (89%-99%)	0.842

A	rP1-C	27	7	50% (37%-63%)	92% (86%-98%)	0.775

A	rAtpD-rP1-C	31	8	57% (44%-71%)	91% (89%-99%)	0.842

A	Ani Labsystems	46	38	85% (77%-95%)	56% (45%-66%)	0.801

G	rAtpD	42	3	78% (67%-89%)	97% (93%-100%)	0.943

G	rP1-C	37	9	69% (56%-81%)	90% (83%-96%)	0.869

G	rAtpD-rP1-C	43	5	80% (69%-90%)	94% (89%-99%)	0.925

G	Ani Labsystems	52	61	96% (91%-100%)	29% (19%-39%)	0.663

^a^Children infected by *M. pneumoniae.*

^b^Healthy blood donors.

**Table 3:** Performance of the rAtpD, rP1-C ELISAs and the Ani Labsystems kit in adults

Ig class	Type of test	No. of positive sera in		Sensitivity (95% CI)	Specificity (95% CI)	AUC
		Patients^a ^(49)	Controls^b ^(86)			
M	rAtpD	33	8	67% (54%-80%)	91% (85%-97%)	0.877

M	rP1-C	22	9	45% (31%-59%)	90% (83%-96%)	0.708

M	rAtpD-rP1-C	39	7	80% (68%-91%)	92% (86%-98%)	0.891

M	Ani Labsystems	24	7	49% (35%-61%)	92% (81%-97%)	0.685

A	rAtpD	32	5	65% (52%-78%)	94% (89%-99%)	0.894

A	rP1-C	27	9	55% (41%-69%)	90% (83%-96%)	0.779

A	rAtpD-rP1-C	36	9	73% (61%-86%)	90% (83%-96%)	0.841

A	Ani Labsystems	48	38	98% (94%-100%)	56% (45%-66%)	0.803

G	rAtpD	30	3	61% (48%-75%)	97% (93%-100%)	0.877

G	rP1-C	22	9	45% (31%-59%)	90% (83%-96%)	0.708

G	rAtpD-rP1-C	33	1	67% (54%-80%)	99% (97%-100%)	0.891

G	Ani Labsystems	48	61	98% (94%-100%)	29% (19%-39%)	0.734

^a^Adults infected by *M. pneumoniae.*

^b^Healthy blood donors.

Serum samples from 39 (72%) children and 24 (49%) adults were IgM-positive based on the Ani Labsystems ELISA. The IgA and IgG Ani Labsystems EIA assays showed the best sensitivity for serum samples from both children and adult patients, with IgA being detected in 46 (85%) children and 48 (98%) adults and IgG being detected in 52 (96%) children and 48 (98%) adults (Tables 2 and 3). It should be noted that although the IgM Ani Labsystems showed good specificity for children and adults (92%), its specificity for IgA and IgG were much lower, at 56% and 29%, respectively (Tables 2 and 3). Indeed, 44% (38/86) and 71% (61/86) of the blood donor serum samples were found to be positive by the IgA and IgG Ani Labsystems commercial kits, respectively (Tables 2 and 3).

For the three ELISA tests, a significant increase in IgM, between two- and three-fold, was detected between the first (acute-phase serum) and second of the six paired serum samples. A two-fold increase in the IgA and IgG responses was also seen between the first and second samples (data not shown).

### Statistical analysis of the rAtpD - rP1 combination

**Figure 3:**
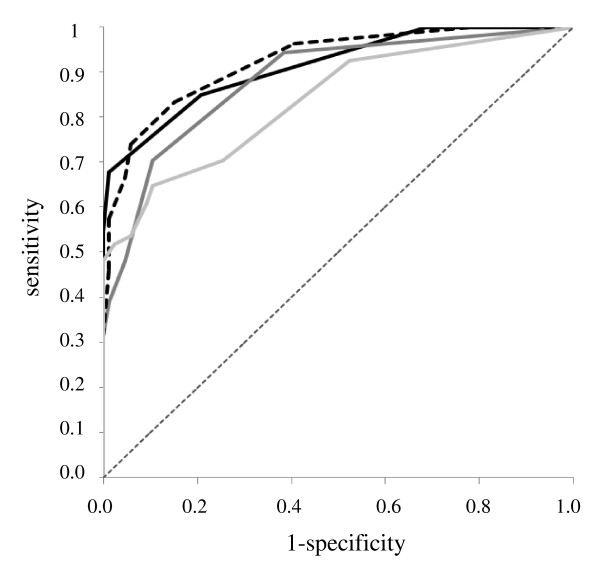
**ROC analysis of the IgM rAtpD, rP1-C ELISAs, alone or combined, and the Ani Labsystems kit in children**. ROC curves for each assay. Black line represent rAtpD (AUC = 0.923), dark gray line rP1-C (AUC = 0.897), black dotted line rAtpD-rP1-C combination (AUC = 0.925), light gray line Ani Labsystems (AUC = 0.824), gray dotted line median (AUC = 0.5).

**Figure 4:**
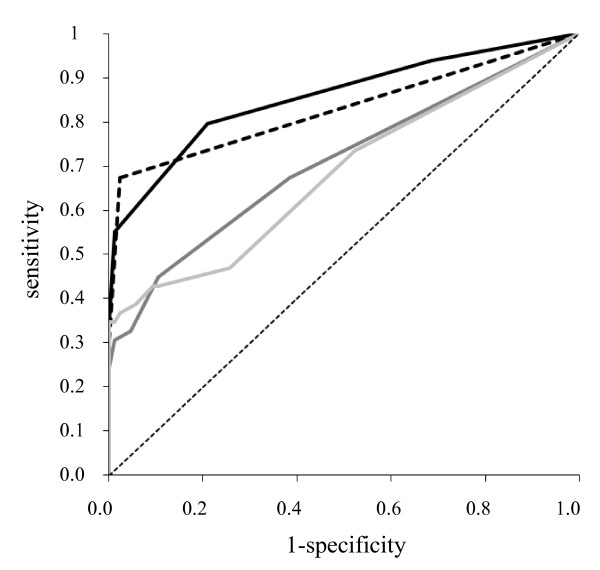
**ROC analysis of the IgM rAtpD, rP1-C ELISAs, alone or combined, and the Ani Labsystems kit in adults**. ROC curves for each assay. Black line represent rAtpD (AUC = 0.877), dark gray line rP1-C (AUC = 0.708), black dotted line rAtpD-rP1-C combination (AUC = 0.891), light gray line Ani Labsystems (AUC = 0.685), gray dotted line median (AUC = 0.5).

To evaluate whether a combination of multiple antigens increased the ability to distinguish patients with RTIs from healthy donors, we compared the seroreactivity with a single antigen (rAtpD or rP1-C protein) to a combination of these antigens and to a P1-enriched total extract from the Ani Labsystems kit (Tables 2 and 3, Fig. 3 and 4).

The AUC score increased with the number of recognised antigens, going from 0.854 for a single recognised antigen to 0.925 for two recognised antigens for IgM class in children and from 0.708 to 0.923 for the IgM class in adults. Moreover, the AUC scores of the combination of the IgM class were higher in children and adults than the respective scores seen with the Ani Labsystems kit (Tables 2 and 3, Fig. 3 and 4).

The rAtpD - rP1-C ELISA IgM combination showed the best sensitivity, detecting 40 (74%) and 39 (80%) of the serum samples from infected children and adults, respectively, compared with the sensitivity obtained with the recombinant antigens alone or the P1-enriched total extract from the Ani Labsystems kit (Tables 2 and 3, Fig. 3 and 4). Combination of the two antigens primarily improved the IgM sensitivity for adult serum samples (Table 3, Fig. 4).

The best sensitivity for the detection of IgG and IgA in serum samples from children and adults sera was obtained with the Ani Labsystems ELISA using P1-enriched total extract. Nonetheless, with regard to specificity, no more than 10% (9/86) of the blood donor serum samples were detected positive for IgA and IgG by the recombinant protein combination, contrasting with the 44% (38/86) and 71% (61/86) found to be positive for IgA- and IgG, respectively, with the Ani Labsystems kit (Tables 2 and 3).

### Cross-reaction studies

We preliminarily evaluated the specificity of the rAtpD ELISA-based assay for IgM, IgA and IgG with a panel of 55 serum samples from patients with non-*M. pneumoniae *RTIs including *Chlamydia pneumoniae *(n = 18), *Legionella pneumophila *(n = 10), *Coxiella burnetii *(n = 10), *Streptococcus pneumoniae *(n = 8), *Bordetella pertussis *(n = 8) and *Chlamydia psittaci *(n = 1). The rAtpD ELISA assay showed good specificity (≥ 94.5%) for all three antibody classes, with no more than 3 of the 55 serum samples cross-reacting (Table 4).

**Table 4:** Cross-reactivity study with the IgM, IgA and IgG rAtpD recombinant protein-based ELISA tests

	No. of sera with false-positive results by the rAtpD ELISA assay for		
Sera from patients infected with (no. of sera tested)	IgM	IgA	IgG

*C. pneumoniae *(18)	3	0	0

*L. pneumophila *(10)	0	0	0

*C. burnetii *(10)	0	1	0

*S. pneumoniae *(8)	0	2	0

*B. pertussis *(8)	0	0	0

*C. psittaci *(1)	0	0	0

## Discussion

Respiratory disease due to *M. pneumoniae *can be assessed by serological methods, and of these the CFT and ELISA are most widely used. The conserved C-terminal region of the P1 adhesin (rP1-C) was recently confirmed as the main antigen for the immunodiagnosis of *M. pneumoniae *infections [13,16].

This work reports the first immunoproteomic study for *M. pneumoniae*, leading to the identification of new antigenic proteins such as the ATP synthase beta subunit, enolase, the pyruvate dehydrogenase beta subunit (PDH-B) and fructose bisphosphate aldolase. Antibodies against the GroEl protein have previously been reported in serum samples from patients with RTIs [24]. All of the antigens described in this study, except the enolase protein, were previously described as "proteins of the Triton X-100 insoluble fraction of *M. pneumoniae*" [25]. These proteins may be associated or bound to a cytoskeleton-like structure, which could provide the necessary framework to maintain and stabilize the shape of *M. pneumoniae *[26], to allow motility [27] and to allow the formation of an asymmetric cell. The correct assembly of this organelle is a prerequisite for the binding of *M. pneumoniae *to specific receptors on the host cell [28,29]. Previous studies have demonstrated that the enolase and the PDH-B protein in addition to their major biosynthetic and metabolic roles in the cytoplasm, could be translocated to the surface to serve as plasminogen- and fibronectin-binding proteins, respectively, facilitating interactions between mycoplamas and the extracellular matrix [30,31]. Thus, these data suggest a pivotal role for these proteins in the infection mechanism of *M. pneumoniae*.

Serologic proteome analysis showed that the AtpD and the P1 proteins were highly detected by serum samples from patients with RTIs and not from healthy blood donors. The other proteins identified were less able to discriminate between patients and controls as they were lightly antigenic to blood donors (confirmed with further ELISA studies, data not shown). Thus the AtpD and the rP1-C proteins were selected for further serological study focusing on comparisons of the performance of assays using these recombinant proteins with assays using adhesin P1-enriched total extracts such as the commercial Ani Labsystems kit.

To this end, the *atpD *gene and the P1-C sequence were cloned, expressed in *E. coli*, and purified. The serological performance of the two recombinant proteins either alone or in combination (logistic regression analysis), and of the Ani Labsystems kit were further compared using a panel of 103 serum samples from *M. pneumoniae*-infected patients (54 children and 49 adults) and 86 serum samples from healthy blood donors. The rAtpD protein-based assay, either alone or in combination with rP1-C, identified more patient serum samples (children or adults) than the Ani Labsystems test for IgM, but fewer than the Ani Labsystems EIA tests for IgA and IgG. In particular, 80% of the serum samples from infected adult were found to be IgM-positive by the combination of the two antigens compared with 70%, 44% and 48% by rAtpD alone, rP1-C alone and the Ani Labsystems assay, respectively (Table 3). Previous studies have shown that young people tend to have higher level of IgM antibodies in acute infections, while adults may lack IgM during this phase [7]. In recent studies, however, most of the IgM assays tested showed inaccurate sensitivity ranging from 30 to 80% [8,32]. Thus the good sensitivity of the rAtpD - rP1-C combination, especially in adults, seems promising and could be suitable for a rapid IgM assay [33].

When studying responses of healthy blood donors, the rAtpD or rP1-C or rAtpD-rP1-C based assay detected a few sera positive for IgM, IgA and IgG. In contrast, a high number was detected positive with the IgA and IgG-EIA Ani Labsystems assays. Such a high IgG seroprevalence in the control serum samples has been observed in previous studies with the same kit [8,12], suggesting the possibility of false-positive results for that assay. The evaluation of the performance of IgG assays, however, is complicated by the lack of information on previous *M. pneumoniae *infections for the control serum samples. As described in a previous study of the prevalence of *M. pneumoniae *IgG and IgA antibodies in a healthy population [34], the seroprevalence increases with age but doesn't exceed 58% for IgG or 28% for IgA, even among the ederly. The elevated levels of specific *M. pneumoniae *IgG antibodies may be caused by past *M. pneumoniae *infections [32,35]. In addition, a variety of non specific antibodies may develop in association with *M. pneumoniae *infection due to the sequence homology of adhesin proteins and glycolipids of the *M. pneumoniae *cell membrane with mammalian tissues [7,12].

The IgA and IgG assays using recombinant proteins (alone or in combination) may lack sensitivity compared to the results obtained with the commercial assay. Nonetheless, the use of recombinant proteins may be more specific than the whole extract used in the Ani Labsystems assays, avoiding the detection of cross-reactive antibodies to *M. pneumoniae*. Many studies have reported the advantage of using a purified recombinant protein in serodiagnosis arguing that better defined antigen preparations should give more accurate results and should be more specific than the use of a glycolipid or whole-cell antigen [17,36,37].

Preliminary cross-reactivity studies were performed to assess the specificity of the rAtpD ELISA assay and showed weak cross-reactivity with other organisms involved in respiratory disease, including *S. pneumoniae*, *C. pneumoniae *and *C. psittaci*, *L. pneumophila*, *B. pertussis *and *C. burnetii*. Three serum samples from *C. pneumoniae*-infected patients and two serums samples from *S. pneumoniae*-infected patients were detected by the rAtpD assay. This could have been due to antigenically similar epitopes, but we could not exclude the possibility of co-infection as these serum samples were found to be positive by the rP1-C assay, as well (data not shown). These data suggest a low risk of cross-reactivity of this assay with an immune response to other respiratory tract bacterial infections, but more RTI serum samples should be tested to confirm these results. The false-positive results could be also explained by cross-reactivity between the rAtpD proteins of *M. pneumoniae *and *M. genitalium*, a phylogenetically closely related species to *M. pneumoniae*. However, we were not able to collect and study some serum samples from *M. genitalium*-infected patients as the diagnosis of these infections is only based on molecular methods. It would be very interesting to further include some serum samples from *M. genitalium*-infected patient in the study.

## Conclusion

In summary, this study presents a new antigen, AtpD, that could contribute to improvements in the diagnosis of *M. pneumoniae *infection in the early and acute phase and could be more specific than the commercial assays using complex extracts. We have shown that the combination of rAtpD with rP1-C antigen to detect IgM contributed to improvements in the early specific diagnosis of *M. pneumoniae *infection. Indeed, several studies have recently reported that combination of selected antigens provide higher sensitivity than single antigens [38].

## Methods

### Organisms and growth conditions

The *M. pneumoniae *reference strain M129 (ATCC 29342) was cultured in SP4 medium containing phenol red used as pH indicator. Tissue culture flasks (Nunc) were incubated at 37°C and inspected daily for colour changes. The exponential growth phase was indicated by a colour change in medium from red to orange-yellow. The cells were harvested at this stage and washed in phosphate buffered saline (PBS) and the pellet was stored at -20°C.

### Patients and healthy blood donors

From January 2004 to December 2006, serum samples were retrospectively selected from 103 patients (54 children, 1-15 years of age and 49 adults, 17-82 years of age), admitted to Pellegrin hospital (Bordeaux, France), Cochin hospital (Paris, France), and Raymond Poincaré hospital (Garches, France) with a diagnosis of *M. pneumoniae *RTI. All of the serum samples were found to be positive for *M. pneumoniae *by serodiagnosis, along with a positive direct diagnosis for some patients, with either culture or PCR. Depending on the laboratory where the *M. pneumoniae *serodiagnosis was done, the serological methods used were either the CFT (Virion Antigen) and a commercial IgM ELISA test (Platelia EIA, Bio-Rad, ImmunoCard *Mycoplasma *Test, Meridian) or a combination of IgM and IgG ELISAs (ImmunoWell IgM, IgG EIA, BMD). Six paired serum samples were collected with an acute-phase sample and a convalescent sample obtained at least two weeks after the first sample. A control group consisting of 86 single serum samples from healthy adult blood donors included in the study was obtained from the French establishment of blood (Rungis, France) and diagnosed as *M. pneumoniae*-negative by the CFT and an IgM ELISA test (Platelia, Bio-Rad). Furthermore, the specificity of the rAtpD protein-based ELISAs was assessed using 55 additional serum samples, 18 that were positive for a *C. pneumoniae *infection (National Reference Center for *Chlamydiae*, Université Victor Segalen Bordeaux 2, France), 10 that were positive for a *L. pneumophila *infection (National Reference Center for *Legionella*, Université Lyon 1, France), 10 that were positive for a *C. burnetii *infection (Pellegrin hospital, Bordeaux, France), 8 that were from patients harboring a *S. pneumoniae *RTI (Raymond Poincaré hospital, Garches, France), 8 that were positive for a *B. pertussis *infection *(*Marcel Merieux Laboratory, Lyon, France), and 1 that was positive for a *C. psittaci *infection (National Reference Center for *Chlamydiae*, Université Victor Segalen Bordeaux 2, France). The present project is in compliance with the Helsinki Declaration (Ethical Principles for Medical Research Involving Human Subjects). The study was done in accordance with the guidelines of the ethical committees of the participating hospitals. In each hospital, specimens were collected as part of the routine management of patients without any additional sampling, and patients provided no objection for their samples to be used. According to the French legacy, this study did not need to be examined by the French "Comité pour la Protection des Personnes" and allowed the exemption of patient's written informed consent. All patient data shown in the present work were anonymously reported, without offering any possibility to trace the actual patients.

### 2D-E

The bacterial pellet were suspended in rehydratation solution (Ready-Prep 2-D Rehydratation/Sample Buffer 1, Bio-Rad) composed of 7 M urea, 2 M thiourea, 1% (wt/vol) ASB-14 detergent, 40 mM Tris, 4% 3[(3-cholamidopropyl)-dimethylammonio]-1-propanesulfate (CHAPS), 0.2% (vol/vol) immobilised pH gradient (IPG) buffer, pH 3-10, 20 mM dithiothreitol (DTT) and 0.002% bromophenol blue. Cell lysis was performed by sonication three times 20 s (Branson Sonifier), and the un-disrupted cells were removed by centrifugation (20,817 × g; 45 min; 21°C). Total protein concentration was determined using a 2-D Quant kit (GE Healthcare) according to the manufacturer's instructions. The protein concentration was calculated using bovine serum albumin (BSA) as a standard.

Isoelectric focusing was performed using the Protean IEF Cell system and Immobilised pH gradient (IPG) strips with a pH range of 5-8 (Bio-Rad). Two hundred and fifty μg of the protein samples in 150 μl of rehydratation solution was used to rehydrate the IPG strips (7 cm, pH 5-8) overnight at 20°C under mineral oil. The proteins were focused for 10 kVh with a maximum voltage of 4,000 V at 20°C. After focusing, the strips were incubated for 10 min in 2 mL of equilibration buffer I (6 M urea, 2% SDS, 375 mM Tris-HCl, pH 8.8, 20% glycerol, 130 mM DTT), followed by equilibration buffer II (6 M urea, 2% SDS, 375 mM Tris-HCl, pH 8.8, 20% glycerol, 135 mM iodoacetamide). The equilibrated IPG strips were then drained and embedded on top of 12.5% acrylamide gels, and electrophoresis was carried out at 25 V for 20 min, and at 180 V for 6 h. Protein molecular weight markers (Bio-Rad) were used. Proteins were visualised by staining with Coomassie blue. Gel images were captured by a GS-800 densitometer (Bio-Rad). Replicate gels were generated from two independent experiments, and one representative gel is shown. The control immunoblot was incubated with the secondary antibody without any human serum and failed to yield any signal.

### Western blot

For western blot analysis, the proteins separated by electrophoresis were transferred to nitrocellulose membranes (0.45 μm, Bio-Rad) [39] and blocked in Tris-buffered saline (TBS) containing 3% non-fat dry milk. The membranes were probed with anti-*M. pneumoniae *antibody-positive pooled human serum or healthy blood donor pooled serum (*n *= 10) at a dilution of 1:500 in blocking buffer. The blots were washed with TBS containing 0.05% Tween 20 (TBST). Goat anti-human alkaline phosphatase conjugate (Sigma-Aldrich) was used as secondary antibody (1:2,000 dilution). Blots were then incubated with *p*-nitroblue tetrazolium chloride and 5-bromo-4-chloro-3-indolyl phosphate *p*-toluidium salt (BCIP) solution (Sigma-Aldrich) until colour development had reached the desired level (2 to 3 min).

### Protein identification and mass spectrometry

Selected protein spots were excised from the 2D-E gels using a sterile scalpel and placed into 96 well plates. The gel pieces were further subjected to in-gel tryptic digestion as previously described [40] with minor modifications. The gel pieces were washed three times in MilliQ water, dehydrated in acetonitrile, and dried in a vacuum centrifuge. They were then rehydrated at 4°C for 15 min in digestion buffer containing 50 mM NH_4_HCO_3 _and 12.5 ng/μl trypsin (modified sequencing grade, Promega). The supernatant was replaced with 30 μl of 50 mM NH_4_HCO_3_, and the samples were incubated overnight at 37°C. Digested peptides contained in the supernatant were purified using a home-made micropurification column containing 0.1 μl of 20R2 reversed-phased material (PerSeptive Biosystems) packed in a Gel-Loader tip (Eppendorf) and equilibrated with 1% trifluoroacetic acid. Ten μl of the supernatant was then loaded onto the column. After washing with 1% trifluoroacetic acid, the adsorbed peptides were eluted directly onto a MALDI target (MTP AnchorChip 600/384, Bruker Daltonik) with 0.8 μl of 70% acetonitrile and 0.1% trifluoroacetic acid containing saturated alpha-cyano-4-hydroxycinnamic acid (Bruker Daltonik). Mass fingerprints were acquired with a MALDI-TOF/TOF mass spectrometer (Ultraflex; Bruker Daltonik) and processed with FlexAnalysis 2.2 software and ProteinScape 1.3 (Bruker Daltonik). After internal calibration with trypsin autodigestion peptides, the monoisotopic masses of the tryptic peptides were used to query NCBInr sequence databases (215, 9330197 sequences) using the Mascot search algorithm (Mascot server version 2.2; http://www.matrixscience.com). The search conditions used were as followed: maximum mass error of 70 ppm, one missed cleavage allowed, modification of cysteines by iodoacetamide, and methionine oxidation as variable modification. Identifications were based on the MASCOT score, observed pI and mass (kDa), number of matching peptide masses and total percentage of the amino acid sequence covered by the peptides. Sequence coverage ranged from 16% to 80%.

### PCR amplification, cloning and expression of the atpD gene and the C-terminal fragment of the p1 gene (rP1-C) of *M. pneumoniae* M129

Sequence cloning was done using the Gateway^® ^technology. This technology allows the efficient transfer of DNA fragments into plasmids while maintaining the reading frame, using a set of recombination sequences, "Gateway att" sites, and two enzymes termed LR Clonase and BP Clonase. Recombination sequences must be introduced to the DNA fragments before cloning into Gateway^® ^vectors. Genomic DNA was extracted from *M. pneumoniae *M129 with the DNA easy tissue kit (Qiagen) and used as a template for PCR amplification of the *atpD *gene (*mpn598*, nucleotide positions 5'-719548-720975-3' on the complementary strand) and the C-terminal fragment of the *p1 *gene (*mpn141*) encompassing amino acid residues 1159-1519 (nucleotide positions 5'-184335-185418-3'). No codon changes were required for expression of the sequences in *E. coli*. The following forward and reverse primers were used for the amplification of the *atpD *gene: 5'-AAAAAAGCAGGCTTGAAAAAGGAAAACATTACATACG-3' (F_a_) and reverse 5'-AGAAAGCTGGGTTTTCTCCTCAACAGTAG-3' (R_a_). The following forward and reverse primers were used for the amplification of the *p1 *gene: 5'-AAAAAAGCAGGCTTGCGGCCTTTCGTGGCAGTTG-3' (F_p_) and reverse 5'-AGAAAGCTGGGTGGTCACTGGTTAAACCGGAC-3' (R_p_). The 13 and 12 first nucleotides of forward and reverse primers, respectively, represented the first recombination sequence necessary for Gateway^® ^cloning. Other nucleotides of the Fa, Ra and Fp, Rp primers represent *atpD *and *p1 *sequences, respectively. PCR was performed in a 25-μl reaction containing 0.075 U/μl of Triple Master polymerase (Eppendorf), 2.5 μl of High Fidelity Buffer with Mg^2+^, 200 μM dNTPs, 200 nM of each primer and 70 ng of extracted DNA. The reaction conditions were standardised at an initial denaturation of 94°C for 5 min followed by 25 cycles of 94°C for 50 s, 54°C for 50 s, and 72°C for 1 min 20 s. A final extension was done at 72°C for 5 min. PCR products were analysed in a 1% agarose gel and purified using a QIA-quick PCR purification kit (Qiagen). A second PCR using the same conditions was performed to introduce the second recombination sequence necessary for Gateway^® ^cloning by using 5'-GGGGACAAGTTTGTACAAAAAAGCAGGCT-3' (F_g_) as the forward primer and 5'-GGGGACCACTTTGTACAAGAAAGCTGGGT-3 (R_g_) as the reverse primer. After purification, the PCR products were inserted into the Gateway^® ^expression vector pDEST17, as previously described [41]. The inserts were then sequenced to rule out any mutations.

The ligation mixtures were transformed into *E. coli *DH5α competent cells. Transformants were selected on LB plates containing 100 μg/ml ampicillin, and the positive clones were confirmed by colony PCR with the F_g _and R_g _primers. Plasmid DNA was isolated from positive clones from overnight cultures using a Midi plasmid purification kit (Qiagen). Fifty ng of plasmid DNA was transformed into *E. coli *BL21 (DE3), and cells containing the recombinant plasmids were grown in 17 ml of LB broth (containing 100 μg/ml ampicillin and 20 μg/ml chloramphenicol) to an optical density at 600 nm (OD_600_) of 0.3. Protein expression was induced by 0.5 mM IPTG (isopropyl-D-thiogalactopyranoside, Sigma-Aldrich). Once the OD_600 _had reached 1, the bacteria were pelleted by centrifugation and further subjected to SDS-PAGE as described by Laemmli [42] and western blot to evaluate the expression and antigenicity of the expressed recombinant proteins. The two proteins were found to be expressed in inclusion bodies. The expression protocol was specifically designed to increase the quantity of expressed recombinant proteins in order to facilitate further purification. Bacterial growth was monitored by measuring absorbance at 600 nm. No toxic effect due to the over-expressed recombinant proteins was observed on *E. coli *cells.

### Purification of recombinant rAtpD and rP1-C proteins

For large-scale production of recombinant proteins, 2l of culture of *E. coli *cells expressing rAtpD and rP1-C were grown and induced with 0.5 mM IPTG. After induction, the bacterial pellet was obtained by centrifugation at 5,251 × g for 6 min at 4°C and resuspended in 60 ml of lysis buffer (20 mM Tris HCl, pH8, 0.5 M sucrose, 100 mM EDTA, pH8, 2 mg/ml lysozyme, 1 mM phenylmethylsulfonyl fluoride (PMSF)). After incubation on ice for 45 min, the tubes were centrifuged at 15,557 × g at 4°C for 10 min. The pellets were frozen at -20°C until purification. The cells were then sonicated three times with a 20 s pulse at 1-min intervals on ice in a sonication buffer (8 M urea, 20 mM triethanolamine, pH8, 500 mM NaCl, 25 mM imidazole, 1 mM PMSF). The cells were harvested by centrifugation at 15,557 × g for 45 min with a buffer containing 20 mM triethanolamine, pH8, 500 mM NaCl and 0.25 M imidazole and then subsequently using buffers with the same composition containing 1 M and 8 M urea. These three "wash steps" were used to eliminate the majority of *E. coli *contaminants before purification. The supernatant of the final step containing the protein of interest was filtered and loaded onto a HisTrap column (GE Healthcare) at 4°C. The proteins were eluted from the column by applying a gradient of 0.25 to 0.5 M imidazole in a buffer containing 8 M urea, 20 mM triethanolamine, pH8, 500 mM NaCl. Fractions containing the recombinant protein in large quantities without contaminants were pooled and dialyzed against an ion exchange buffer (6 M urea, 20 mM triethanolamine, pH8) overnight using a nitrocellulose dialysis membrane (Spectra/Por^®^membrane kit, http://www.spectrumlabs.com) before loading onto a HiTrap ion exchange Q column (GE Healthcare). The proteins were eluted by applying a gradient of 0 to 1 M NaCl in ion exchange buffer. The fractions containing the recombinant proteins with a high degree of purity were pooled and dialyzed against a storage buffer (6 M urea, 20 mM triethanolamine, pH8, 300 mM NaCl, 5 mM EDTA). The protein concentration was determined by the Lowry method [43]. The fractions were separated by 12.5% SDS-PAGE and the purity of purified recombinant proteins was estimated by densitometry (Quantity one software, GS 800 densitometer, Bio-Rad). The purified proteins were instantaneously used for ELISA analysis, the proteins were then conserved no longer than one month in storage buffer.

### ELISAs with purified recombinant proteins rAtpD, rP1-C and commercial Ani Labsystems kit

Serum samples collected from children and adult patients with *M. pneumoniae *RTIs and from healthy blood donors were screened for anti-*M. pneumoniae *IgM, IgA and IgG antibodies by in-house ELISAs with the rP1-C and rAtpD proteins. Preadsorption of IgG rheumatoid factor was performed before each IgM ELISA test. The purified proteins were diluted by successive steps in PBS to avoid potentially damaging crystallisation of the urea in our ELISA washer automates. No precipitation of proteins was observed. Control ELISA tests were performed at different urea concentrations ranging from 8 M to 0.1 M. The reactivity of the two recombinant proteins was not affected by stepwise dilution as the variation of the ELISA values with control serum samples was insignificant. The 96-well Maxisorp microtitre EIA plates (Nunc) were coated in triplicate with 50 ng per well of rP1-C or rAtpD in PBS. The plates were incubated overnight at 4°C and blocked in 250 μl blocking buffer (4% bovine serum albumin in PBS with 5 mM EDTA) at 37°C for 1 h. After washing three times with PBS containing 0.05% Tween 20, the antigen-coated wells were incubated sequentially for 30 min at 37°C with 1:100-diluted test sera, along with 1:50,000 dilution of peroxidase-labelled goat anti-human IgM, or IgA, or a 1:200,000 dilution of peroxidase-labelled goat anti-human IgG (Pierce). Plates were washed three times with PBS containing 0.05% Tween 20 between incubations. The enzyme reaction was developed with 100 μl of TMB (tetramethylbenzidine) substrate (Medac) for 30 min at 37°C. The reaction was stopped by adding 100 μl of 2 M H_2_SO_4_. The plates were read by photometric reading at 450 nm using an Opsys MR microplate reader (Dynex). "Blank" wells containing no patient or control serum were included in each assay. No high background was observed (OD_450 _≤ 0.05). Panels of serum samples from 103 patients and 86 healthy blood donors were screened for anti-*M. pneumoniae *IgM, IgG and IgA antibodies using the corresponding Ani Labsystems EIA kits according to the manufacturer's instructions.

### Statistical analysis

All results were analysed with the Tanagra software 1.4.31 (http://chirouble.univ-lyon2.fr/~ricco/tanagra/fr/tanagra.html). The accuracy of the serological assays in discriminating disease cases from normal cases was evaluated by using ROC curve plots [44]. ROC plots were calculated by expressing the relationship between the fraction "correctly identified to be positive" and the fraction "falsely identified to be positive" for every possible cut-off point selected to discriminate between the patients and the blood donors. The AUC is a measure of the assay efficiency to discriminate the "true positives" from the "true negatives". The cut-off values for every in-house serological assay were determined for maximum efficiency of the test. A sample was considered positive if the antibody titre exceeded the defined cut-off value. Binary logistic regression analysis was performed before evaluating the performance of the antigen combination by ROC plots as described above. Sensitivity, specificity and 95% confidence intervals (95% CI) were calculated for rAtpD and rP1-C antigens, either alone or in combination. The calculation of cut-off values and the interpretation of the results of the Ani Labsystems kits were performed according to the manufacturer's instructions.

## Authors' contributions

HN performed experiments, analysed the data, and wrote the manuscript. CC participated in designing the experiments and analysing the data. HR performed experiments. SP selected the patient serum samples and participated in analysing the data. CB participated in designing the experiments, and analysing the data, and wrote the manuscript. All of the authors read and approved the final manuscript.
